# PineappleDB: An online pineapple bioinformatics resource

**DOI:** 10.1186/1471-2229-5-21

**Published:** 2005-10-05

**Authors:** Richard L Moyle, Mark L Crowe, Jonni Ripi-Koia, David J Fairbairn, José R Botella

**Affiliations:** 1 Plant Genetic Engineering Laboratory, Department of Botany, University of Queensland, QLD 4072, Brisbane, Australia; 2 Institute of Molecular Biosciences, University of Queensland, QLD 4072, Brisbane, Australia; 3 Department of Biological Science, University of Waikato, Hamilton, New Zealand

## Abstract

**Background:**

A world first pineapple EST sequencing program has been undertaken to investigate genes expressed during non-climacteric fruit ripening and the nematode-plant interaction during root infection. Very little is known of how non-climacteric fruit ripening is controlled or of the molecular basis of the nematode-plant interaction. PineappleDB was developed to provide the research community with access to a curated bioinformatics resource housing the fruit, root and nematode infected gall expressed sequences.

**Description:**

PineappleDB is an online, curated database providing integrated access to annotated expressed sequence tag (EST) data for cDNA clones isolated from pineapple fruit, root, and nematode infected root gall vascular cylinder tissues. The database currently houses over 5600 EST sequences, 3383 contig consensus sequences, and associated bioinformatic data including splice variants, *Arabidopsis *homologues, both MIPS based and Gene Ontology functional classifications, and clone distributions. The online resource can be searched by text or by BLAST sequence homology. The data outputs provide comprehensive sequence, bioinformatic and functional classification information.

**Conclusion:**

The online pineapple bioinformatic resource provides the research community with access to pineapple fruit and root/gall sequence and bioinformatic data in a user-friendly format. The search tools enable efficient data mining and present a wide spectrum of bioinformatic and functional classification information. PineappleDB will be of broad appeal to researchers investigating pineapple genetics, non-climacteric fruit ripening, root-knot nematode infection, crassulacean acid metabolism and alternative RNA splicing in plants.

## Background

In terms of commercial production, pineapple [*Ananas comosus *(L.) Merrill] is the third most important tropical fruit after banana and mango. Pineapple fruits are classified as non-climacteric, as there is no respiratory burst or spike in ethylene production during ripening and exogenous application of ethylene does not rapidly accelerate fruit ripening. Much has been learnt about the control of fruit ripening in climacteric fruit using tomato as a model system. In particular, manipulation of genes involved in the ethylene biosynthetic pathway and a MADS box transcription factor have led to altered ripening characteristics [1-5]. Conversely, almost nothing is known of how non-climacteric fruit ripening is controlled. Efforts to identify genes controlling non-climacteric fruit ripening are hampered by the small number of non-climacteric fruit gene sequences available for study. Thus, as a first step toward understanding the molecular basis of non-climacteric fruit ripening in pineapple, an EST sequence project has been initiated to isolate expressed sequences from mature green unripe and yellow ripened pineapple fruits [6].

Many crop species, including pineapple, are susceptible to root-knot nematode infection. Crop losses due to nematode infections are estimated to be more than 100 billion dollars each year [7]. Additionally, the toxic soil fumigants used to control nematodes are becoming increasingly banned in many countries. Understanding the molecular mechanisms governing the nematode-plant interaction is of utmost importance in developing alternative strategies for the control of nematode infection. As such, we have constructed EST sequencing libraries from pineapple root and gall vascular cylinder tissue infected with the root-knot nematode *Meloidogyne javanica*. The vascular cylinder contains the giant cell structures that the nematode feeds upon, and can be dissected from the root cortex and stripped of nematodes with relative ease. Sequencing EST clones from such libraries is a first step toward isolating and identifying gene sequences involved in the plant-nematode interaction.

The collection of EST sequence information requires accurate gene annotation as well as dedicated platforms for storage, processing, curation and data retrieval. Ideally, collected sequence information should be easily accessed, and presented in a user-friendly format that provides the tools to mine the data efficiently. PineappleDB was developed to provide the research community with access to a curated and searchable bioinformatics resource housing fruit, root and gall derived EST sequences, contig sequences, clone annotations, functional classification and Gene Ontology information – all via a user-friendly web interface. PineappleDB will be a valuable resource of broad appeal to researchers studying pineapple genetics, crassulacean acid metabolism, non-climacteric fruit ripening, alternative RNA splicing in plants and root-knot nematode infection.

## Construction and content

### Database architecture

The database was developed using MySQL 4.0, and implemented on a server running RedHat 9.0. The web interface uses cgi scripts written in Perl 5.8.1. Perl scripts were also used for data processing and uploading into the database. Field descriptions and a full database schema are provided on the help page of the website.

### PineappleDB web interface

The pineapple bioinformatic resource can be accessed through a web interface [8]. The introductory page contains information about the pineapple bioinformatic resource, access to the pineapple EST database, lists of contigs containing full-length coding sequences, alternatively spliced clones, putative nematode sequences, and links to pineapple related web pages.

**Figure 1:**
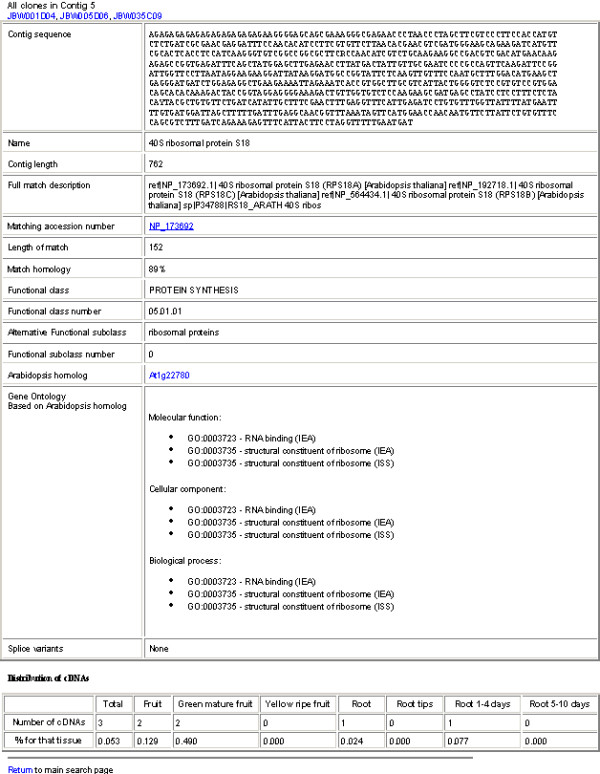
Example of contig search output information from PineappleDB.

The pineapple EST sequence database can be searched by cloneID, contigID, text, or by sequence homology to either individual EST sequences or contig consensus sequences. Both BLASTN and TBLASTX searches can be performed against the pineappleDB with multiple searches also possible. The search output contains clone and contig information, including sequence, putative identification, a link to the nearest homologue in the NCBI nr database, length of homologous sequence and percentage identity of nearest match, a link to information on the closest homologue in *Arabidopsis*, MIPS based functional classifications, Gene Ontology information, and the presence of splice variants (Fig. 1). Links to all EST clones clustering within the same contig, and the distribution of the EST's across the fruit and root libraries are also listed in the search output.

PineappleDB will be periodically upgraded as annotation, functional classification, and GO information are updated.

## Utility

### EST sequencing and bioinformatic analysis pipeline

Users may refer to the pineappleDB flow diagram on the homepage of the website for an overview of the bioinformatics pipeline. In total, 7296 clones from five libraries were 5' end sequenced. The libraries were constructed from uninfected root tips (~2 cm), dissected vascular cylinders of galls from early infection (1–4 weeks post infection), dissected vascular cylinders of galls from late infection (5–10 weeks post infection), mature green fruit and mature yellow fruit. Over 75% of clones returned an average Phred20 score of more than 700 bp. Raw sequences were manually edited for sequence quality and trimmed of plasmid contaminant and polyA tail in the sequence viewer program Chromas v2.13 (Technelysium). 5861 edited sequences were retrieved at an average read length of 769 bp. The 1615 clones with poor sequence quality and/or yielding less than 150 bp of insert sequence were eliminated from further bioinformatic analysis.

The 408 green fruit, 1140 yellow fruit, 343 root tip, 1298 early infection and 2461 late infection edited EST sequences were clustered into 3383 contigs, using SeqMan sequence assembly software (DNASTAR Inc. Madison, USA) and key parameters of minimum 90% match over at least 45 bp overlap. All edited EST sequences have been submitted to the GenBank dbEST [GenBank: CO730741-CO732287, DT335767-DT339792] [9]. Each sequence was assigned a putative identification by BLASTX alignment of contig consensus sequences to the GenBank non-redundant (nr) protein database [10]. Those clones that did not retrieve a BLASTX match better than the 10^-20 ^E-value cut-off were annotated as an undiscovered sequence. The sequences were also BLASTX searched against MATDB to retrieve putative MIPS based functional classifications for clones with a homologous gene from the model plant organism *Arabidopsis thaliana *[11]. GO classifications were obtained by downloading GO results from a multiple search of the TAIR *Arabidopsis *resource [12]. A semi-automated process of parsing BLAST hits and manually curating the putative annotations resulted in a spreadsheet of information including cloneID, contig number, number of clones in each contig, nearest BLASTX match, accession number of match, length of match, percent similarity, putative annotation and functional classifications.

### Identification of full length clones

Contig consensus sequences containing a polyA tail were analyzed for open-reading frames using EditSeq sequence analysis software (DNASTAR Inc. Madison, USA) and were BLASTX searched against the GenBank nr protein database [13]. Contig consensus sequences were identified as containing a putative full length coding sequence by alignment to known full-length protein coding sequences, and/or by the presence of stop codons upstream of a significant open reading frame. A list of the putative full length coding sequences identified is present in the pineapple bioinformatic resource.

### Identification of splice variants

Edited clone sequences generally assembled into contigs with 97–100% homology. However, the contig assembly report occasionally revealed incidences where some clones clustered with between 90–97% homology or that some clones did not cluster into existing contigs due to homology somewhat below the 90% threshold. An inspection of these clone sequences and contigs revealed the presence of apparently unspliced intron sequence, and/or the absence of exon sequence in some of the clones. A comparative analysis to other clone sequences within the contig alignment and to homologous protein coding sequences in GenBank verified that 120 clones contain an apparent "mis-splicing" event. The putative splice variant clones containing un-spliced intron sequence and/or missing spliced exon sequence are listed in the pineapple bioinformatic resource. The presence or absence of a putative splice variant is also reported in contig/clone search outputs.

### Identification of putative nematode sequences

Despite precautions to remove nematodes from root tissues prior to library construction, it was anticipated that there would be some contamination of the pineapple gall libraries with nematode derived sequences. All contig consensus sequences containing root and gall EST's were BLASTN searched against the GenBank dbEST and BLASTX searched against the GenBank nr database. Matches to known nematode sequences were manually inspected and 77 contigs identified as containing a putative nematode sequence. All contigs containing putative nematode sequence are listed within the online pineapple bioinformatic resource.

## Conclusion

The pineapple EST sequencing project was initiated as a first step toward identifying genes involved in and the molecular basis of non-climacteric fruit ripening and the nematode-plant interaction. The online pineapple bioinformatics resource was developed to house EST sequence information and associated bioinformatic data in a user-friendly format. PineappleDB can be freely accessed via the internet, and currently contains BLAST and text search tools to efficiently mine the dataset for clones and contigs of interest. The resulting search outputs contain comprehensive information on the clone and contigs including cloneID, contig number, number of clones in each contig, nearest BLASTX match, accession number of match, length of match, percent similarity, putative annotation, splice variants, MIPS based functional classifications, Gene Ontology classifications, and the distribution of clones from each library (fig. 1). Links are also provided to other clones within the same contig, the GenBank BLASTX nearest neighbour, and to homologous coding sequences from the model organism *Arabidopsis thaliana*.

PineappleDB houses the first reported collection of EST sequences isolated from pineapple. PineappleDB will grow as more EST sequence information becomes available. Furthermore, we have initiated a pineapple microarray project and it is anticipated that gene expression data will be incorporated into the online pineapple bioinformatics resource in the future. The EST database will periodically be upgraded as annotation, functional classification, and gene ontology information is updated.

## Availability and requirements

The PineappleDB resource can be accessed via 

Contact: Dr. José R Botella at j.botella@uq.edu.au

## Authors' contributions

*The authors wish it to be known that, in their opinion, the first two authors should be regarded as joint first authors. RM was responsible for data collection, the bioinformatic pipeline and manuscript preparation. MC developed the online database and undertook batch BLAST processes. JR-K contributed to the fruit EST editing and identification of full length coding sequences. DJF participated in the conception, design and co-ordination of the study and helped complete the manuscript. JRB designed, supervised and coordinated the project.
